# Editorial: Endocrine aspects of gynaecological cancers, volume II

**DOI:** 10.3389/fendo.2025.1659320

**Published:** 2025-07-31

**Authors:** Stefano Palomba

**Affiliations:** ^1^ Obstetrics and Gynecology, University Sapienza of Rome, Rome, Italy; ^2^ Grande Ospedale Metropolitano of Reggio Calabria, Reggio Calabria, Italy

**Keywords:** endocrinological oncology, cancer, malignancies, gynaecological, editorial

A new interesting area of research regards the relationship between hormonal factors and gynaecological cancers. More and more preclinical data and clinical evidence underline that the development, progression, and management of gynaecological malignancies may be influenced by hormones, even if that interplay is very intricate and complex.

The current Research Topic titled “*Endocrine aspects of gynaecological cancers, volume II*” offers to the readers invaluable insights into this critical area of research and clinical practice. After an intense selection and refereeing process five articles were included in this Research Topic that quickly surpassed 3000 hits at the time of writing this editorial.

The Gjorgoska et al.‘s manuscript is an *in vitro* study aimed to assess 11-oxyandrogen formation from steroid precursors and its bioactivity profile in endometrial cancer cell lines of different differentiation degree and molecular subtype having as control a cell line of normal endometrium. 11-oxyandrogens are specific androgen metabolites produced by adrenal glands and characterized by an oxygen atom at C11 position. Contrary to classic androgens, serum 11-oxyandrogens levels remain consistent in post-menopause and may play a role in physiological and pathological processes during the post-reproductive female period. The pre-clinical data obtained by Gjorgoska et al. demonstrate that the formation of 11-oxyandrogens in endometrial cancers does not occur from classic androgen precursors and that 11-oxyandrogens have distinct regulatory mechanisms according to the tumour grading with higher levels of bioactive 11-keto-testosterone in low-grade endometrial cancers in comparison with normal endometrium and high-grade, TP53 mutated endometrial cancers. This study supports the role played by androgens in development and progression of the endometrial cancers suggesting that serum 11-oxyandrogens levels may be a useful prognostic biomarkers in patients with endometrial cancers.

Xiang et al. performed a systematic review with meta-analysis aimed to assess the risk of ovarian cancer associated in women who received hormone replacement therapy (HRT). Data were analysed according to study design, study period, treatment duration, and different regimens. Twenty-one cohort and 30 case-control studies including very large populations were analysed. After data synthesis from cohort studies, the pooled risk of ovarian cancer for HRT was 20% higher in users versus non-users. A slightly lower but significant risk of ovarian cancer (13% with a range from 4% to 22%) was detected after meta-analysis of data from case-control studies. Of note, when only more recent data were analysed, the risk disappeared, suggesting that new formulations and protocols are probably safer in comparison with those used in the past. As expected, the risk increased with prolonged exposure time (particularly for durations exceeding 10 years), and no difference was detected between combined and sequential use of estro-progestins and between oestrogens and estro-progestins.

Absolute or relative hypoestrogenism has been considered an important risk factor the development of uterine fibroids and some cancers. Zhao et al. studied the causal associations between uterine leiomyoma and 16 site-specific cancers using a two-sample Mendelian randomization analysis using the public genome-wide association studies datasets. That study revealed an association between uterine leiomyomas and ovarian, breast and brain cancers. In particular, the risk of malignancies varied from 7% to 29% in patients with uterine fibroids. The ovarian cancers mainly involved were the low malignant potential, and the serous, invasive mucinous, and clear cell ovarian cancers. On the other hand, uterine fibroids were associated with a little but significant reduction of the risk of gastric cancer. Even if the precise underlying mechanisms remain to be fully elucidated, the overall effect of uterine leiomyomas was consistent in an elevated risk of malignancies suggesting that the affected patients could benefit from preventive strategies for the early detection of cancers.

Ferroptosis is a specific mode of cell death driven by the accumulation of iron-dependent lipid hydroperoxides that intertwine with metabolism, redox-state and biology, and pathological processes. In addition, ferroptosis in the tumour microenvironment plays a pivotal role in the regulation of infiltrating immune cells and in their crosstalk with tumour cells. The capping actin protein, gelsolin-like (CAPG), also known as Macrophage Capping Protein, regulates cell motility by remodelling actin filaments, which participate in cell migration and invasion in several types of cancers. CAPG is a potential therapeutic target in various cancers, and its high expression may be related to ferroptosis activation, as demonstrated in hepatocellular carcinoma. On the contrary, its potential immunotherapeutic effects and prognostic value in endometrial cancer is poorly known. To this regard, Liu et al. performed a preclinical study and clarified the relationship between CAPG and endometrial cancer showing as this protein may modulate the immune response and ferroptosis in endometrial cancer too suggesting that CAPG may be a new immunotherapy target for patients with uterine cancer.

Oxidative stress has been suggested as a crucial factor influencing carcinogenesis in endometrial cancer and the use of statins may be a potential intervention to reduce the cellular redox-state with specific actions on several enzymes. Based on these premises, Ollila et al. conduced a retrospective study on patients with type 2 diabetes who were diagnosed with endometrial cancer under statin treatment. The expression of redox-state regulating proteins nuclear factor erythroid 2 related factor 2 (Nrf2) and Kelch-like ECH-associated protein 1 (Keap1) in the tumour samples were assessed immunohistochemically, and manganese superoxide dismutase (MnSOD) levels were assessed both immunohistochemically in neoplastic tissue and in serum samples. Patients with similar characteristics that did not use statins were used as controls. They found that only the expression levels of Keap1 and Nrf2, but not MnSOD, were different between statin users vs. non-users. High MnSOD expression predicted better progression-free survival in statin non-users at univariate analysis with Kaplan Meier curves. However, at multivariate Cox regression analysis, the redox-state regulating proteins were not found to be associated with overall survival or progression-free survival after adjusting data for stage and statin use.

In conclusion, the present Research Topic includes important and interesting preclinical and clinical papers that confirm the scientific interest for the onco-endocrinology and the need for further research into the endocrinology of gynaecological cancers.

